# pH-activatable brominated pentamethine cyanine dyes for imaging-guided photodynamic immunotherapy of tumors

**DOI:** 10.1039/d6sc02842j

**Published:** 2026-05-13

**Authors:** Wangna Tang, Yanbing Cao, Ran Wang, Tian Qiu, Yingqi Hu, Shaoyang Shi, Xiaolong Zeng, Jiangli Fan, Wen Sun, Xiaojun Peng

**Affiliations:** a State Key Laboratory of Fine Chemicals, Frontiers Science Center for Smart Materials Oriented Chemical Engineering, Dalian University of Technology Dalian 116024 China sunwen@dlut.edu.cn yanbingcao@dlut.edu.cn; b Ningbo Institute of Dalian University of Technology Ningbo 315016 China

## Abstract

Photodynamic therapy (PDT) has emerged as a minimally invasive antitumor strategy, yet conventional photosensitizers often suffer from off-target phototoxicity under physiological conditions. This study presents a novel pH-activatable brominated pentamethine cyanine dye (C-CyBr) as a smart photosensitizer for imaging-guided photodynamic therapy (PDT). A hydroxylated indole moiety was rationally introduced as the pH-sensitive switch. Under neutral physiological pH (7.4), the dye exists in a closed-ring non-fluorescent and photoinert state with negligible reactive oxygen species (ROS) generation and fluorescence emission. In contrast, once exposed to the acidic tumor microenvironment (pH ≤ 6.5, the critical activation threshold), the hydroxyl group undergoes rapid protonation, which restores the conjugated π-electron system, activates fluorescence imaging signals and simultaneously unlocks the photosensitizing activity. C-CyBr was further used to prepar nanoparticles (OCBr), which exhibit excellent tumor accumulation *via* the enhanced permeability and retention effect. Following tumor accumulation, the fluorescence signal of OCBr enables real-time monitoring of its biodistribution, thereby guiding the optimal timing for light irradiation. Subsequently, OCBr undergoes endocytosis into cancer cells, where the acidic lysosomal environment (pH < 5.0) triggers nanoparticle disassembly and release of the active photosensitizer. The liberated photosensitizer then escapes from lysosomes and specifically targets mitochondria, enabling localized photodynamic damage upon light irradiation. *In vivo* experiments reveal that OCBr-mediated PDT not only strongly inhibits tumor growth but also elicits immunogenic cell death, promoting dendritic cell maturation and CD4^+^/CD8^+^ T-cell infiltration, thereby activating a systemic antitumor immune response. OCBr serves as a promising pH-activatable theranostic platform for PDT with boosted efficacy and immunomodulatory potential.

## Abstract 2

This pH-activatable C-CyBr dye is photoinert under physiological conditions and turns on fluorescence/PDT in acidic tumors. Corresponding nanoparticles realize imaging-guided therapy to inhibit tumor growth and induce systemic antitumor immunity.

## Graphical abstract

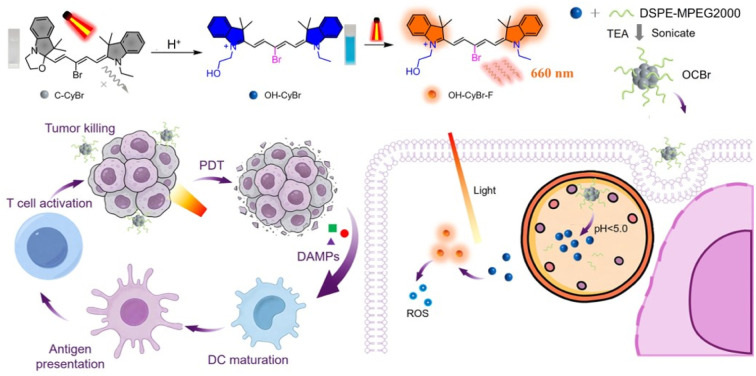

## Introduction

Photodynamic therapy (PDT) has emerged as a minimally invasive and spatially selective treatment modality for various cancers, which relies on the combination of photosensitizers, light, and oxygen to generate cytotoxic reactive oxygen species (ROS) that induce tumor cell death. Compared with conventional treatments such as surgery and chemotherapy, PDT offers advantages including reduced systemic toxicity, repeatable applicability, and the potential to stimulate antitumor immunity.^1–3^ Despite its promise, the clinical translation of PDT is often hindered by the limitations of conventional photosensitizers, including poor tumor selectivity and high dark toxicity. Furthermore, many existing photosensitizers exhibit constant “always-on” fluorescence and phototoxicity, which can lead to unintended damage to normal tissues and reduce imaging contrast during therapy.^4^ Therefore, the development of activatable photosensitizers that remain inert until reaching the tumor microenvironment has become a key strategy to enhance therapeutic specificity and safety.

Cyanine dyes, have attracted considerable attention as theranostic agents due to their tunable optical properties, ease of structural modification, and good biocompatibility. Especially, pentamethine cyanines activated by near-infrared (NIR) light enable deep tissue penetration, while also offering high brightness, making them suitable for both imaging and PDT.^5–7^ However, their inherent dark toxicity, insufficient ROS quantum yield, and lack of tumor-specific activation remain major challenges that limit their therapeutic efficacy. To address these issues, molecular engineering strategies have been explored. Introducing heavy atoms such as bromine or iodine can promote intersystem crossing (ISC) *via* the heavy-atom effect, thereby enhancing ROS generation.^8,9^ Meanwhile, rational functionalization with pH-labile groups enables fluorescence and phototoxicity to be switched on specifically in the acidic tumor microenvironment, improving tumor-to-normal tissue contrast.^10–14^

**Scheme 1:**
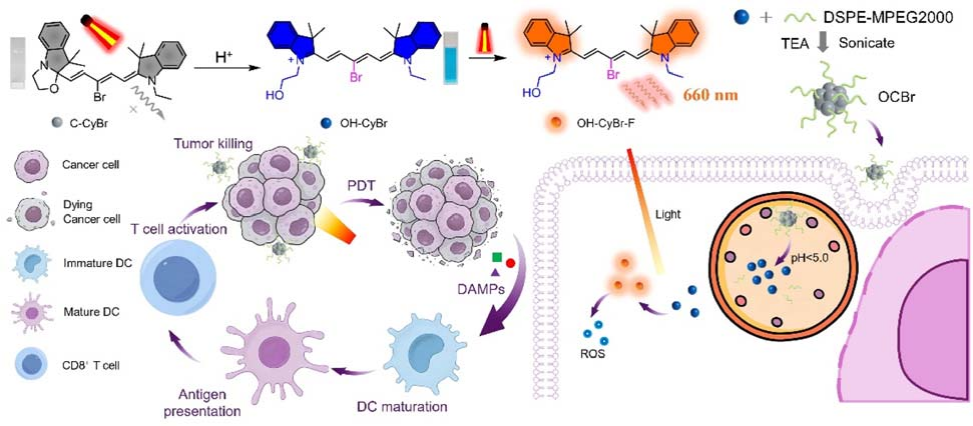
The chemical structure of C-CyBr and the pH-activatable mechanism; schematic illustration for-the preparation of OCBr and its anticancer process under NIR light activation.

In this study, we designed and synthesized a novel acid-activatable brominated pentamethine cyanine dye, designated C-CyBr, which integrates pH-responsive fluorescence switching and reactive oxygen species (ROS) generation. In the molecular design, including bromination at the *meso*-position boosts intersystem crossing and ROS production, and hydroxyethyl functionalization on the indole moiety is able to improve aqueous solubility and confer pH responsiveness, which enables selective ring-opening in the acidic tumor microenvironment, accompanied by fluorescence turn-on and therapeutic activation. C-CyBr was then co-assembled with DSPE-MPEG2000 *via* triethylamine (TEA) mediated co-assembly to form nanoparticles (OCBr), which accumulate in tumors through the enhanced permeability and retention (EPR) effect.^15–17^ In the acidic tumor microenvironment, the nanoparticles facilitate the release of C-CyBr, which undergoes a pH-responsive ring-opening transformation to form OH-CyBr. This activated species further targets mitochondria after cellular internalization. Upon light irradiation, OCBr generates abundant ROS to induce mitochondrial damage and immunogenic cell death, accompanied by the release of damage-associated molecular patterns including CRT and HMGB1. These signals promote dendritic cell maturation and antigen presentation, thereby activating CD4^+^ and CD8^+^ T lymphocytes and eliciting a robust antitumor immune response, as verified in murine tumor models.^18,19^ This integrated theranostic strategy that unites tumor microenvironment-responsive activation, enhanced photodynamic therapy, and immunomodulation, providing a promising candidate for next-generation precision phototherapy (Scheme 1).

## Results and discussion

### Synthesis and pH-responsive spectral properties of C-CyBr

In order to prepare C-CyBr, the brominated pentamethine cyanine dye (OH-CyBr) was firstly successfully synthesized *via* a two-step condensation reaction (Scheme S1) and fully characterized by ^1^H NMR, ^13^C NMR, ESI HRMS (Fig. S1–S3, SI), and UV vis spectroscopy. To obtain the pure closed-form product, a small amount of base was added dropwise to the solution of OH-CyBr, inducing the formation of the oxazolidine ring and causing C-CyBr to precipitate from the solution. The structural transformation was confirmed by ^1^H NMR analysis, which revealed distinct chemical shift changes characteristic of the closed form (Fig. S4). Notably, under physiological conditions (*e.g.*, in PBS at pH 7.4), the hydroxyethyl group on the indole moiety spontaneously undergoes cyclization with the adjacent carbonyl to form the oxazolidine ring, yielding the closed form C-CyBr.

**Fig. 1:**
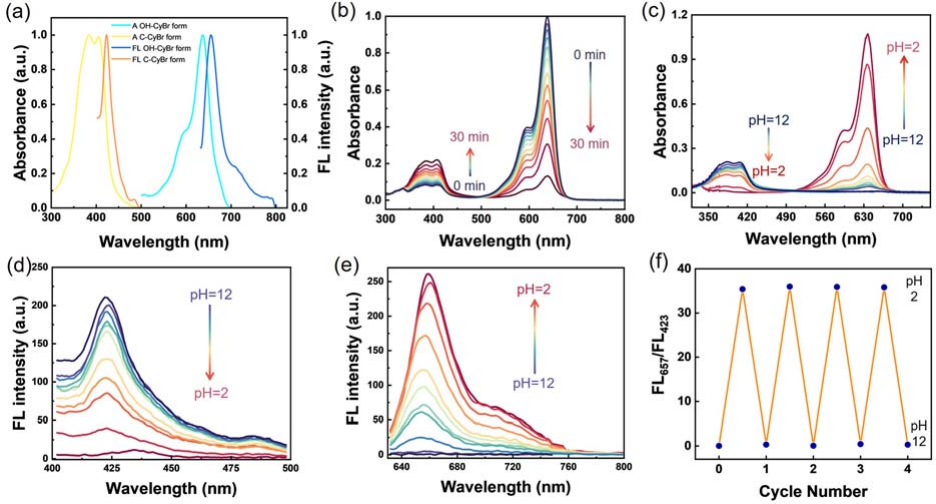
pH-responsive spectral properties. (a) Normalized absorbance and fluorescence plot of C-CyBr and OH-CyBr (2.5 µm) state in DCM. (b) UV-vis absorption spectra of OH-CyBr (2.5 µm) in PBS solution within 30 minutes. (c) UV-vis absorption spectra of OH-CyBr (2.5 µM) in buffers with pH ranging from 2.0 to 12.0 (pH = 2, 3, 4, 5, 6,7, 8,9,10, 11,12). (d and e) pH-dependent fluorescence spectra of OH-CyBr (2.5 µM) in buffer solutions (pH 2.0–12.0) with excitation at (d) 380 nm and (e) 660 nm, respectively. (f) Reversible fluorescence OFF/ON switching upon pH cycling between two fixed endpoints (pH 2 and pH 12) for four cycles.

C-CyBr (oxazolidine form) exhibits a maximum absorption wavelength at 408 nm with an almost colorless appearance, and its maximum emission wavelength is located at 423 nm. Under basic and physiological pH conditions, the dominant oxazolidine form, which possesses a shorter π-conjugated system, displays a maximum absorption peak at 408 nm in the visible region and is nearly colorless. In contrast, when the pH is below 6.4, the system mainly exists as the ring-opened structure OH-CyBr, which shows a maximum absorption wavelength at 635 nm and a maximum emission wavelength at 655 nm. Single and sharp absorption and emission bands are observed in the UV-vis absorption and fluorescence spectra, indicating the formation of pure pentamethine cyanine dye without by-products (Fig. 1a). In the PBS solution environment (Fig. 1b), the absorbance at 638 nm decreases while that at 408 nm increases within 30 minutes. This phenomenon indicates that the side-chain cyclization process disrupts the conjugated system, resulting in a change in the absorption spectrum, which reflects the corresponding structural transformation of the probe over time. Under acidic pH, the formation of the highly conjugated OH-CyBr structure, which appears blue, leads to the emergence of an absorption peak at 638 nm. Concurrently, the peak at 408 nm decreases, and a distinct isosbestic point is observed at 500 nm (Fig. 1c). By fitting the absorbance ratio at 638 nm and 408 nm (Fig. S5), the pH-responsive transition point was determined to be pH = 4.36, which matches the acidic environment of lysosomes (pH < 5.0). This suggests that the probe has the potential for specific response within cancer cells. In alkaline solution, deprotonation of the hydroxyethyl group attached to the indolium moiety generates an oxyanion nucleophile. This nucleophile then attacks the electron-deficient iminium carbon *via* 5-endotrig cyclization, forming a fused oxazolidine ring. Consequently, reversible pH-dependent shifts in both absorption and emission spectra are observed. The fluorescence emission maxima differ markedly between the closed oxazolidine compound and the open OH-CyBr form. Fluorescence pH titrations were performed in buffered solutions over a range of pH values, with the probe concentration held at 2.5 µM. Under basic conditions, the oxazolidine compound exhibits an emission peak at 425 nm when excited at 380 nm. As the pH decreases during titration, the visible-wavelength emission (*λ*_em_ = 425 nm, *λ*_ex_ = 380 nm) progressively loses intensity (Fig. 1d). Simultaneously, a new NIR emission band (*λ*_em_ = 660 nm, *λ*_ex_ = 635 nm) turns on and increases correspondingly, which is attributed to the formation of the highly conjugated open OH-CyBr structure (Fig. 1e). Furthermore, the probe exhibited wonderful reversibility with fast response when the pH was regulated back and forth between two fixed endpoints (pH = 2 and pH = 12) at least four cycles (Fig. 1f). To assess potential interference, various biologically relevant analytes were tested. Fluorescence measurements of the pH-activatable probe were carried out at 37 °C and pH 7.4 in the presence of key metal ions (Na^+^, K^+^, Ca^2+^, Mg^2+^, *etc.*), anions (Cl^−^, HCO_2_^−^, NO_2_^−^, *etc.*), and bioactive small molecules including glutathione, cysteine, and H_2_O_2_ (Fig. S6).^20^

### Theoretical calculation and *in vitro* evaluation of photodynamic performance

**Fig. 2:**
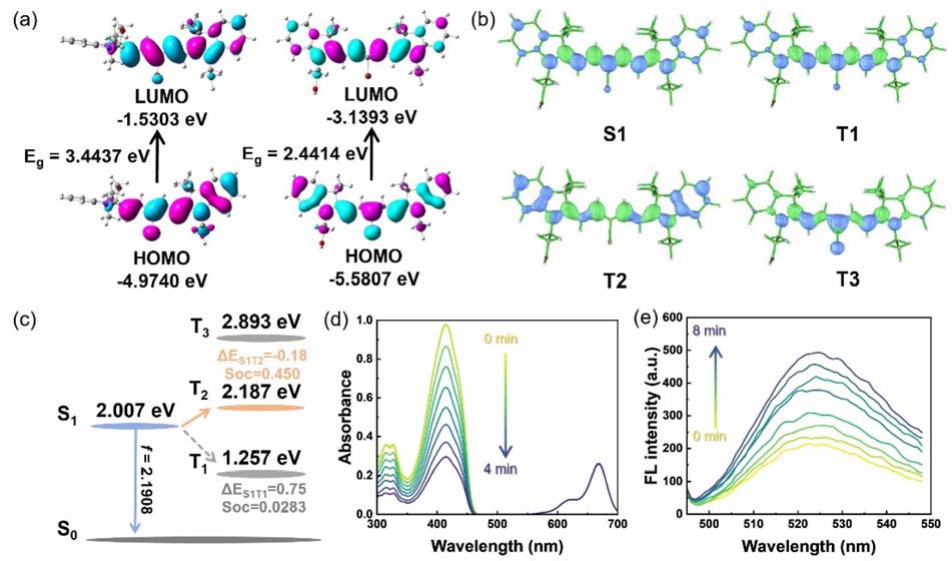
Quantum chemical analysis of the PDT performance and electronic structure. (a) HOMO/LUMO surface of geometry-optimized C-CyBr (left) and OH-CyBr (right) in H_2_O. (b) Natural transition orbital distribution of each excited state for OH-CyBr. (c) Schematic of the intersystem crossing formation mechanisms of OH-CyBr determined from TD-DFT calculations. (d) Time-dependent UV-vis absorption spectra of the mixture of OH-CyBr and DPBF in dichloromethane under light irradiation. (e) Time-dependent fluorescence spectra of DHR123 in H_2_O containing OH-CyBr upon light irradiation for different durations.

Given the excellent photophysical properties and unique pH-responsive characteristics described above, we further evaluated the potential application of this photosensitizer in photodynamic therapy (PDT), with a focus on its mechanism and capacity for generating reactive oxygen species (ROS) under light irradiation. The energy level calculation results reveal that, for the open OH-CyBr form, the HOMO energy level (−5.5807 eV) is notably lower than that of the closed oxazolidine compound (C-CyBr) (−4.9740 eV). Correspondingly, its LUMO energy level (−3.1393 eV) is also reduced compared to the closed oxazolidine compound (−1.5303 eV). Consequently, the band gap E_g_ of OH-CyBr is narrowed from 3.4437 eV to 2.4414 eV (Fig. 2a). A smaller band gap implies that the target molecule requires less energy for electronic transition, enabling it to respond to incident light of longer wavelength (635 nm). Meanwhile, the lower LUMO energy level enhances its electron-accepting capacity, which is beneficial for improving the molecule's reactivity in processes such as photocatalysis and photosensitized oxidation.

Natural transition orbital (NTO) analysis helps to paint a clear picture of each excited state (Fig. 2b). For the target molecule, the energy gaps Δ*E*(S_1_–T_1_), Δ*E*(S_1_–T_2_) and Δ*E*(S_1_–T_3_) are determined to be 0.75 eV, −0.18 eV, and 0.886 eV, respectively (Fig. 2c). These values reveal that the energy barrier for intersystem crossing (ISC) is practically negligible (owing to an extremely small energy gap) in the S_1_–T_2_ channel. Conversely, the S_1_–T_1_ gap is moderately large, while the S_1_–T_3_ gap is substantially elevated-thus, ISC is preferentially favored between S_1_ and T_2_. Correspondingly, the spin–orbit coupling (SOC) coefficient for S_1_–T_2_ is 0.450 cm^−1^, which is markedly larger than the 0.0283 cm^−1^ observed for S_1_–T_1_. This indicates a more pronounced SOC effect in the S_1_–T_2_ channel, where the spin-forbidden nature of the transition is significantly mitigated. In contrast, the SOC value of S_1_–T_1_ falls well below the typical threshold for efficient ISC (>0.1 cm^−1^), rendering the spin-forbidden barrier insurmountable. When considering the synergistic influence of energy gap and SOC, the S_1_-to-triplet transition of this molecule is dominated by the S_1_–T_2_ channel, which exhibits both higher ISC efficiency and greater feasibility. By contrast, the S_1_–T_1_ ISC pathway is largely suppressed, as it is constrained by both an unfavorable energy gap and a weak SOC effect.^21^

These favorable photophysical properties lay the molecular-level foundation for efficient reactive oxygen species (ROS) production, which we further validated through solution-phase ROS trapping experiments. 2,3-Diphenylbenzofuran (DPBF), a specific trapping agent for singlet oxygen (^1^O_2_), exhibits a marked reduction in its characteristic absorption peak around 410 nm as irradiation time increases (0 to 4 min), a trend that indicates sustained ^1^O_2_ generation by the photosensitizer under light exposure (Fig. 2d). The mixed solution was irradiated with a 660 nm laser (10 mW cm^−2^) every 30 seconds for a total duration of 240 seconds, and the UV vis absorption spectrum was measured immediately after each irradiation. Concurrently, dihydrorhodamine 123 (DHR123) shows a gradual enhancement in fluorescence intensity around 520 nm over an 8 minute irradiation period (0 to 8 min). This observation confirms that the photosensitizer also produces ˙O_2_^−^*via* the type I photodynamic pathway (Fig. 2e). The mixed solution was irradiated with a 660 nm laser (10 mW cm^−2^) every 30 seconds for a total duration of 240 seconds, and the fluorescence spectroscopy was measured immediately after each irradiation. Collectively, these results demonstrate that OH-CyBr sustains the generation of both ^1^O_2_ and ˙O_2_^−^, thereby exhibiting synergistic type I and type II photodynamic activities.

### Characterization and *in vitro* PDT effects of OCBr

Based on the excellent reactive oxygen species (ROS) generation capability of this photosensitizer *in vitro*, we further formulated it into a nanoscale preparation, with the aim of enhancing its accumulation in tumor sites *via* the enhanced permeability and retention (EPR) effect, and systematically investigated the photodynamic therapy efficacy of this nanosystem at the cellular level.

Initially, direct co-assembly of the ring-opened form OH CyBr with DSPE-mPEG2000 was attempted; however, this approach resulted in suboptimal assembly efficiency, presumably due to incomplete conversion of the photosensitizer to its hydrophobic oxazolidine form (C-CyBr) under neutral aqueous conditions.

**Fig. 3:**
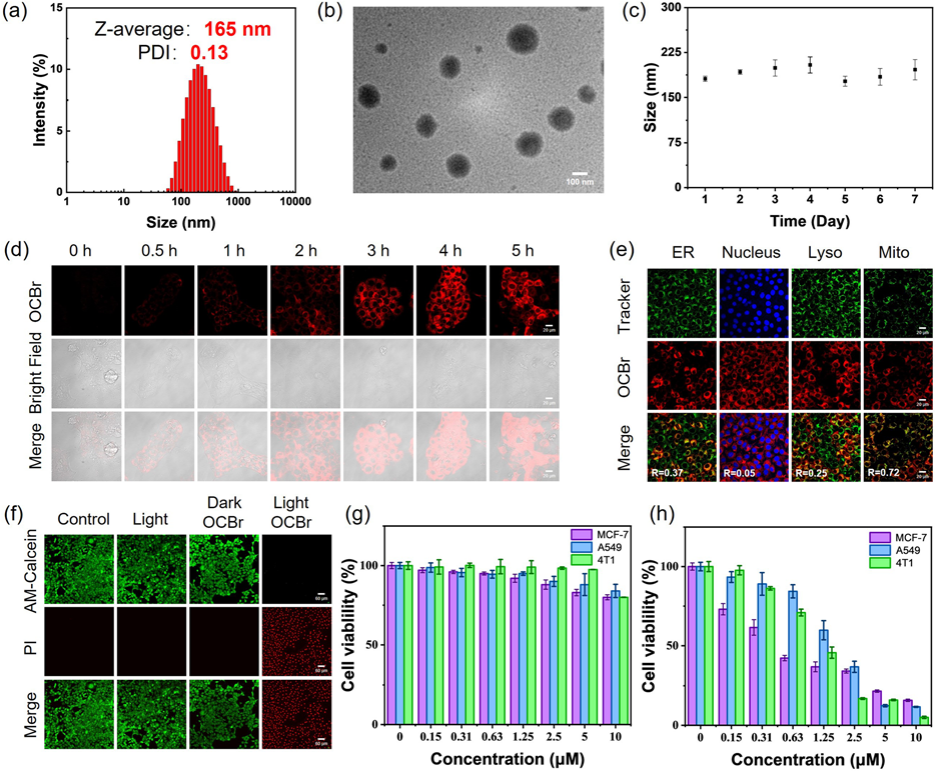
Synthesis and characterization of OCBr and Its *in vitro* cytotoxicity under 660 nm irradiation. (a) Size distribution of OCBr measured by DLS. (b) TEM image of OCBr. Scale bar: 100 nm. (c) Size distribution of OCBr measured over 7 days in PBS. (d) CLSM images of 4T1 cells after incubation with OCBr at different time points. Scale bar: 20 µm. (e) CLSM images of OCBr and its localization in different intracellular organelles (ER, nucleus, lysosome, and mitochondria). Scale bar: 20 µm. (f) Live/dead cell imaging of 4T1 cells after a variety of treatment. Scale bar: 60 µm. Cell viability profiles of M7 (purple), *A*549 (blue), and 4T1 (green) cell lines incubated with the pH-activatable probe at various doses (0.15, 0.31, 0.63, 1.25, 2.50, 5.00, and 10.0 µM) for 24 hours, with (g) or without (h) laser irradiation. Error bars specify standard error of mean (*n* = 5). (2 µM, 660 nm, 20 mW cm^−2^, 10 min).

For better co-assembly, TEA was employed to deprotonate the ring-opened OH-CyBr, shifting it from a hydrophilic state to a hydrophobic oxazolidine form (C-CyBr). Dynamic light scattering (DLS) measurements showed an average hydrodynamic size of 195 nm (Fig. 3a). Transmission electron microscopy (TEM) images revealed that OCBr adopted a spherical morphology with a smooth surface (Fig. 3b), confirming the formation of nanoparticles. After being dissolved in PBS at 37 °C for 7 days, OCBr exhibited no significant change in size, indicating its favorable stability in buffered solution (Fig. 3c). To evaluate the photosensitizer release behavior of OCBr under different pH conditions, *in vitro* release experiments were performed in buffer solutions at pH 6.5 and pH 7.4, and the cumulative release of C-CyBr was determined (Fig. S7 and S8). The release rate of C-CyBr at pH 6.5 (78.1%) was significantly higher than that at pH 7.4 (14.8%). This pH-dependent release profile indicates that the nanosystem exhibits acid responsiveness, which facilitates selective drug release in tumor tissues, thereby enhancing therapeutic efficacy and reducing systemic toxicity. In cellular uptake experiments, fluorescence signals began to emerge within cells at 30 minutes, gradually intensified over time, and stabilized around 4 hours (Fig. 3d). This process clearly illustrates the dynamic distribution and accumulation pattern of OCBr inside cells, indicating that the nanoparticles can enter cells and progressively enrich within them, thereby laying the foundation for subsequent biological functions. In co-localization assays (Fig. 3e), the Pearson's correlation coefficient between OCBr and mitochondria reached 0.72, significantly higher than those with the endoplasmic reticulum, nucleus, and lysosomes, indicating that the photosensitizer predominantly localizes to mitochondria. Next, Calcein-AM and propidium iodide (PI) staining were performed to visualize live and dead cells, respectively. The result of 4T1 cell imaging displayed the green fluorescence of Calcein-AM in the blank and the group of OCBr without light irradiation, indicating that most of the cells remained healthy state. In contrast, in the OCBr group with light irradiation, negligible green fluorescence of Calcein-AM and significant red fluorescence of PI were observed, indicating the almost complete eradication of cancer cells (Fig. 3f).

Furthermore, ROS production was evaluated using fluorescent probes DCFH-DA and DHE. Fluorescence imaging showed that OCBr generated substantial amounts of ROS in 4T1 cells under both normoxic and hypoxic conditions upon light exposure, consistent with the ROS production observed *in vitro* (Fig. S9). AO staining was employed to assess lysosomal integrity. In the control, light-only, and OCBr-treated groups without light, red fluorescence from intact lysosomes was clearly observed. In contrast, after treatment with OCBr under light irradiation, red fluorescence disappeared (Fig. S10), indicating that photoactivated OCBr effectively disrupts tumor cell lysosomes. To confirm that the observed cancer cell death is indeed mediated by ROS, vitamin C (Vc) was used as a ROS scavenger. Upon addition of the ROS scavenger vitamin C (Vc), the DCFH-DA fluorescence signal in the OCBr + light + Vc group was markedly quenched, confirming that the observed cellular effect is indeed mediated by ROS generation (Fig. S11). Cell viability assays using the 3-(4,5-dimethylthiazol-2-yl)-2,5-diphenyltetrazolium bromide (MTT) colorimetric method demonstrated that the probe exhibited low cytotoxicity toward MCF-7, A549, and 4T1 cells under dark conditions. However, a significant difference in cytotoxicity was observed upon light irradiation (Fig. 3g and h).

### *In vivo* immune responses to OCBr-mediated PDT

**Fig. 4:**
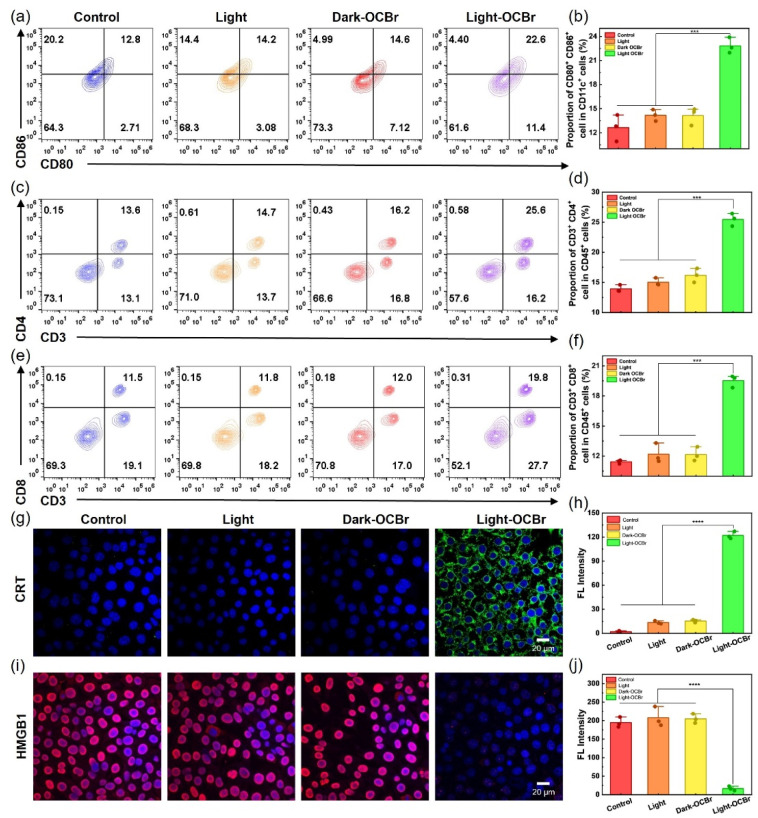
*In vivo* immune response of OCBr-mediated PDT. (a) Flow cytometry analysis of DCs (CD11c^+^CD80^+^CD86^+^) in tumor tissues following different treatment regimens. (b) Corresponding quantitative analysis of mature DCs (*n* = 3). (c) Flow cytometry analysis of helper T cells (CD45^+^CD3^+^CD4^+^). (d) Corresponding quantitative analysis (*n* = 3). (e) Flow cytometry analysis of cytotoxic T cells (CD45^+^CD3^+^CD8^+^). (f) Corresponding quantitative analysis (*n* = 3). (g) Immunofluorescence images and (h) quantitative analysis of calreticulin (CRT) surface exposure. (i) Immunofluorescence images and (j) quantitative analysis of HMGB1 extracellular release in tumor tissues among different treatment groups. Scale bars: 20 µm. Data are presented as mean ± SD (*n* = 3). ***p < 0.001, ****p < 0.0001.

Based on the promising cellular photodynamic efficacy of the nanoscale, we evaluated the photoimmunotherapeutic effects of OCBr. On day 14 of treatment, flow cytometry was used to preliminarily assess the proportions of immune cells in tumor tissues and spleens of mice from the four groups (PBS, light, dark-OCBr, light-OCBr). The percentage of CD80^+^CD86^+^ mature dendritic cells (DCs) in tumor tissues increased from 12.8% to 22.6%, demonstrating that OCBr-mediated PDT enhances antigen release to promote DC maturation (Fig. 4a and b). Mature DCs are critical for promoting the proliferation and differentiation of T cells, thereby augmenting the anti-tumor immune response in mice.

Consequently, we further evaluated the percentages of CD3^+^CD8^+^ and CD3^+^CD4^+^ T cells in tumor tissues. As expected, the light-OCBr group showed significantly higher percentages of CD3^+^CD8^+^ cytotoxic T cells (19.8%) and CD3^+^CD4^+^ helper T cells (25.6%) compared to the OCBr group (11.5% and 13.6%, respectively) (Fig. 4c–f). The expression dynamics of CRT and HMGB1 were additionally examined at tumor sites before and after treatment *via* immunofluorescence staining. As shown in Fig. 4g–j, the light-OCBr group exhibited a significant upregulation of CRT expression on the tumor cell surface, which serves as an “eat-me” signal to recruit and activate antigen-presenting cells, along with a marked downregulation of intracellular HMGB1 expression due to its release into the tumor microenvironment as a pro-inflammatory danger signal. An ATP assay was utilized to assess ATP secretion in order to confirm the ICD induction characteristic of OCBr. According to these findings, the light-OCBr group secreted ATP at a level 4.7-fold higher than that of the PBS group (Fig. S12). These findings indicate that OCBr enhanced ROS generation, leading to increased CRT exposure on the cell surface and promoted release of ATP and HMGB1 from tumor cells, thereby amplifying the ICD-associated immune response.

### *In vivo* antitumor efficacy

**Fig. 5:**
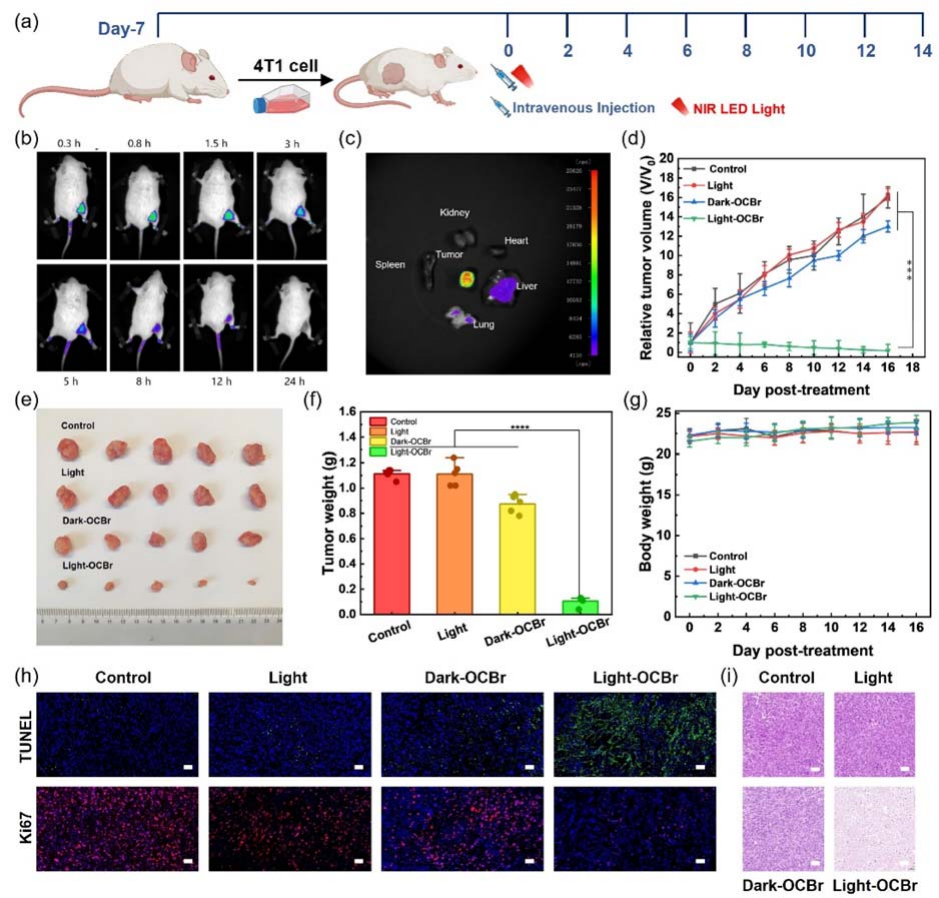
Therapeutic effect of OCBr-mediated PDT in breast cancer mice. (a) Schematic illustration of the *in vivo* phototherapeutic protocol for OCBr *via* tail vein injection. (b) *In vivo* fluorescence imaging of OCBr distribution in mice over time after injection. (c) Fluorescence imaging of dissected tumor, heart, liver, spleen, lung, and kidney in mice after OCBr intravenous injection. Tumor growth curves over time (d), representative photographs of harvested tumors (e), and tumor weights on day 14 (f) in tumor-bearing mice treated with control, light, dark-OCBr, or light-OCBr (660 nm, 50 mW cm^−2^, 10 min). (g) Body weight of the above four groups of mice during 14 days' treatment. (h) TUNEL staining and immunofluorescence images of Ki67 in tumor tissues under different treatment conditions. Scale bars: 50 µm. (i) H&E staining of tumor on day 14 from different treatment groups. Scale bar = 100 µm. (*n* = 5 independent experiments, ns: not significant. ***: *p* < 0.001).

To investigate the antitumor efficacy of OCBr *in vivo*, a tumor-bearing mouse model was established using 4T1 cells. 4T1 cells were implanted in the mice model 7 days prior to PDT (Fig. 5a). Subsequently, all mice received a single-dose injection and a single-light treatment, unless otherwise specified. The mice were grouped into PBS only, PBS with light irradiation, OCBr without light irradiation, and OCBr with light irradiation. *Ex vivo* fluorescence imaging of dissected major organs at 8 h post-administration further confirmed that the fluorescence signal was predominantly localized in the tumor tissue, demonstrating that OCBr possesses excellent tumor-targeting ability (Fig. 5b). Based on these imaging-guided insights, light irradiation was performed at 8 h post-injection to maximize therapeutic efficacy.

After *in vivo* fluorescence imaging of OCBr at 8 h post-administration, the tumor, heart, liver, spleen, lung, and kidney of mice were dissected and subjected to *ex vivo* fluorescence imaging. As observed, the fluorescence signal was predominantly localized in the tumor tissue, demonstrating that OCBr possesses excellent tumor-targeting ability (Fig. 5c and S13). The tumor volume was monitored over time, revealing that the tumor growth in the OCBr with light treatment group ceased after treatment with OCBr under light irradiation (50 µM, 50 mW cm^−2^, 10 min) (Fig. 5d). The relative tumor volumes increased by 13 times after 14 days, indicating that the treatment by irradiation alone or by OCBr only has a negligible influence on tumor growth. To further evaluate the antitumor efficacy of OCBr NPs, the isolated tumors were weighed and photographed after treatment. After 14 days, in the group of OCBr with light irradiation, the tumors were much smaller than those of other groups, implying that effective growth arrest of the tumors was achieved (Fig. 5e).

Meanwhile, the tumor weight in mice from the other groups exhibited rapid growth (Fig. 5f). Furthermore, there was almost no significant body weight loss observed in the mice in each group during the experiment (Fig. 5g), indicating that OCBr exhibited good safety *in vivo*. Correspondingly, a marked enhancement in the signal of TUNEL-positive cells (green fluorescence) was detected in the OCBr-light group compared to the three control groups, indicating significantly enhanced apoptosis in tumor tissues (Fig. 5h). Additionally, Ki67 expression was significantly downregulated in the OCBr-light group relative to the other control groups, which further demonstrates that OCBr-mediated PDT not only induces robust tumor cell apoptosis but also exerts potent anti-proliferative effects, thereby contributing to severe tumor tissue damage.

To further assess the biosafety of OCBr, histological analysis was performed using hematoxylin and eosin (H&E) staining on the major organs of the mice, including the tumor, heart, liver, spleen, lung, and kidney (Fig. S14). As shown in Fig. 5i, obvious cell damage was observed in the tumors of the group treated by OCBr with light irradiation. However, in other groups, no remarkable cell damage was observed. The *in vivo* biocompatibility of OCBr was further assessed. After a 30 day treatment, no statistically significant differences were observed in hematological parameters among the control, light, dark-OCBr, and light-OCBr groups (Fig. S15). Combined with histopathology and serum biochemistry (Fig. S16), these results confirm that OCBr exhibits good biocompatibility at the tested doses.

## Conclusion

In this study, a novel pH-activatable brominated pentamethine cyanine dye, C-CyBr, was designed and synthesized, and further encapsulated into nanoparticles designated as OCBr. Leveraging a pH-responsive mechanism, OCBr achieves fluorescence “turn-on” in the acidic tumor microenvironment, enabling selective tumor imaging. The introduction of a bromine atom enhances intersystem crossing, promoting synergistic type I and type II photodynamic reactions for efficient reactive oxygen species generation. At the cellular level, OCBr exhibits good mitochondrial targeting, low dark toxicity, and potent light-induced cytotoxicity. In animal models, OCBr shows excellent tumor-targeted accumulation and significant tumor growth inhibition upon irradiation. Moreover, OCBr-mediated photodynamic therapy induces immunogenic cell death and enhances antitumor immune responses. OCBr serves as a dual-functional platform for tumor imaging and therapy with favorable biosafety and immunomodulatory potential, offering valuable insights for the development of next-generation theranostic photosensitizers.

## Author contributions

W. T. synthesized and characterized the fluorescent probe, R. W. designed and conducted the biological experiments, T. Q. and Y. H. conducted immunohistochemistry staining and analysis, S. S. and X. Z. supervised the project, and all co-authors wrote and edited the manuscript.

## Conflicts of interest

There are no conflicts to declare.

## Supplementary Material

SC-017-D6SC02842J-s001

## Data Availability

All experimental procedures and associated data are provided in the supplementary information (SI). Supplementary information is available. See DOI: https://doi.org/10.1039/d6sc02842j.
