# Supplementary material for: pH-activatable brominated pentamethine cyanine dyes for imaging-guided photodynamic immunotherapy of tumors
Source: Chem Sci. 2026 May 13;17(25):12597–603. doi: 10.1039/d6sc02842j (PMC13187947; doi:10.1039/d6sc02842j)
Supplement: SC-017-D6SC02842J-s001 [file SC-017-D6SC02842J-s001.pdf]

Supplementary Information (SI) for Chemical Science.
This journal is © The Royal Society of Chemistry 2026

Supporting Information

pH-Activatable Brominated Pentamethine Cyanine Dyes for Imaging-Guided Photodynamic Immunotherapy of Tumors

Wangna Tang,[+a] *Yanbing Cao,**[*a] *Ran Wang,*[a] *Tian Qiu,*[a] *Yingqi Hu,*[a] *Shaoyang Shi,*[a] *Xiaolong Zeng,*[a] *Jiangli Fan,*[a] *Wen Sun,**[*ab] *Xiaojun Peng*[a]

Materials and Methods

1.1. Chemical reagents

2,3,3-Trimethyl-3H-indole, 2-iodoethanol, 2,3-dibromo-2-butenedioic acid, 1-ethyl-2,3,3-trimethyl-3H-indol-1-ium iodide, 1 ethyl 2,3,3 trimethyl 3H indolium iodide, and aniline were purchased from Energy Chemical Co., Ltd. Solvents including acetonitrile, ethanol, diethyl ether, sodium acetate, methanol, ethyl acetate, dichloromethane, N,N-dimethylformamide (DMF), and tetrahydrofuran (THF) were purchased from Xilong Scientific Co., Ltd. Sodium hydroxide, triethylamine (TEA), sodium bicarbonate, and anhydrous sodium sulfate were obtained from Tianjin Damao Chemical Reagent Factory. All solvents were of analytical grade. Ultrapure water was prepared from commercially available Wahaha purified water using a Milli-Q system.

1.2. Experimental instruments

^{1}H NMR and ^{13}C NMR spectra were recorded on a Bruker Advance spectrometer (400 MHz and 500 MHz, Bruker, USA) with tetramethylsilane (TMS) as the internal reference. Mass spectrometry was performed using an Agilent 6320 Ion Trap LC/MS system and a Thermo Scientific TSQ Quantum Ultra triple quadrupole LC-MS/MS system. UV–Vis absorption spectra and fluorescence spectra were recorded on a Perkin Elmer Lambda 35 UV–Vis spectrophotometer and a VARIAN-CARY Eclipse fluorescence spectrophotometer (Serial No. FL0812-M018, Agilent, USA). Dynamic light scattering (DLS) measurements were carried out using a Nano-ZS 90 Nanosizer (Malvern Instruments Ltd., Worcestershire, UK). Transmission electron microscopy (TEM) images were obtained using an HT7700 EXALENS microscope (Hitachi, Japan). Cell viability was determined by measuring absorbance at the corresponding wavelength using a Thermo Fisher microplate spectrophotometer (Serial No. 3020089). Cellular uptake, live/dead cell staining, and colocalization assays were visualized using an Olympus FV3000 confocal laser scanning microscope (CLSM). A 660 nm LED laser source (Shenzhen Guangyuanhong Co., Ltd., China) was used for photodynamic experiments. Flow cytometric analysis was performed using an Attune NxT acoustic focusing cytometer (Thermo Fisher). Light power density was measured using an irradiance meter produced by Beijing Education Au-light Co., Ltd. In vivo fluorescence imaging of mice was conducted using a NightOWL II LB983 small-animal imaging system.

1.3. Biological Reagents

Human lung adenocarcinoma cells (A549), mouse breast cancer cells (4T1), and human breast cancer cells (MCF-7) were purchased from the American Type Culture Collection (ATCC). Cells were cultured in DMEM medium (Gibco, No. C11965500BT). Fetal bovine serum (FBS) was purchased from Meisen Cell Technology Co., Ltd. (CTCC-002-071). Penicillin–streptomycin (KGY0072) and trypsin (KGY0012) were obtained from KeyGen Biotech Co., Ltd. Commercial subcellular organelle trackers (Hoechst 33342, Lyso Tracker Green DND-26, Mito Tracker Green, ER-Tracker Green), an Annexin V-FITC apoptosis detection kit, and a reactive oxygen species (ROS) detection kit (DCFH-DA) were purchased from Beyotime Biotechnology Co., Ltd.

Synthesis of Intermediate Compound 1

2,3,3-trimethyl-3H-indole (10.0 g, 62.8 mmol, 1 eq) and 2-iodoethanol (32.4 g, 188.4 mmol, 3 eq) were dissolved in 30 mL of acetonitrile, and the mixture was stirred under reflux overnight. After the reaction solution was cooled to room temperature, it was filtered, and the solid was washed with ethyl acetate. The resulting pink solid (Compound 1) was directly used in the next reaction without further purification. Yield: 18.7 g (90.1%).

Synthesis of Intermediate Compound 2

2,3-Dibromo-2-butenedioic acid (5.116 g, 20 mmol, 1 eq) was dissolved in 35 mL of ethanol. Subsequently, aniline (3.908 g, 42 mmol, 1 eq) dissolved in 18 mL of ethanol was added dropwise via a constant pressure dropping funnel over 10 min. The reaction vessel was slowly heated to 40 °C with vigorous stirring. In this step, the evolution of CO_2 was used as the indicator for the completion of the reaction: the reaction was deemed incomplete if continuous CO_2 gas evolution was observed. After the reaction was complete, the golden-brown reaction mixture was cooled in an ice bath, and then 50 mL of diethyl ether was slowly added with intense stirring until a bright yellow solid precipitate formed. The solid was filtered, washed with diethyl ether and dried to afford Compound 2 as a golden-brown solid (5.130 g, 85% yield). This solid was directly used in subsequent reactions without further purification.

Synthesis of Intermediate Compound 3

Compound 1 (1.76 g, 5.31 mmol, 1 eq), compound 2 (1.6 g, 5.31 mmol, 1 eq), 1-ethyl-2,3,3-trimethyl-3H-indol-1-ium iodide (1.68 g, 5.31 mmol, 1 eq), and sodium acetate (1.1 g, 13.55 mmol, 3 eq) were dissolved in 25 mL of ethanol and stirred at 80 °C for 2 h. After cooling to room temperature, the mixture was extracted with water and DCM. The organic layers were combined, dried over anhydrous sodium sulfate, and the solvent was removed under reduced pressure. The residue was purified by column chromatography (dichloromethane: methanol = 10: 1) to afford the product OH-CyBr as a dark blue solid (1.56 g, yield 22.4%).

Synthesis of C-CyBr

Compound 3 (50.4 mg, 74.9 μmol) was dissolved in 3 mL of methanol, and approximately 2 drops of 0.5 M aqueous sodium hydroxide solution were added slowly with constant stirring until the blue solution completely precipitated as an orange solid. After cooling to room temperature, the mixture was filtered, and the filter residue was washed with diethyl ether and dried under vacuum to afford the final product C-CyBr. Yield: 42.7 mg (84%).

^{1}H NMR (400 MHz, DMSO-d_6) δ 8.60 (q, J = 8.8 Hz, 4H), 7.62 (d, J = 14.2 Hz, 2H), 7.47 (d, J = 7.5 Hz, 2H), 7.37 (q, J = 7.2 Hz, 4H), 7.19 (t, J = 7.3 Hz, 2H), 6.36 (d, J = 14.2 Hz, 2H), 4.25 (s, 4H), 3.76 (d, J = 5.4 Hz, 4H), 2.72 (s, 4H), 1.91 (s, 2H), 1.33 (d, J = 61.1 Hz, 12H).

ESI-MS: m/z calcd for $[M-I^{-}+H^{+}]^{+}$: 504.18, found: 505.18.

UV/vis Spectroscopy

Absorption spectra were recorded on a Perkin Elmer Lambda 35 UV-Vis with wavelength range from 200 to 1100 nm by a 1 cm path length quartz cuvette. The data were acquired using a PC and processed by manufacturers' supplied Spectra Manager Version 2 software. The absorption ratio signal (I_{638}/I_{408}) was determined from the absorption intensities at 638 nm and 408 nm.

Fluorescence Spectroscopy

Emission traces were obtained on VARIAN-CARY Eclipse, Serial No.FL0812-M018 operated by FluorEssence Version 3.9.0.1 software. 5 nm excitation and emission slit widths were used. The ratio signal (I_{657}/I_{423}) was measured from the fluorescence intensities at 657 nm and 423 nm.

pH Response Experiments

The buffer solutions at various pH were prepared using a Mettler Toledo pH meter. The pH meter was calibrated with the standard buffer solutions of pH 4.01 ± 0.01, 7.00 ± 0.01, and 10.00 ± 0.01 at 25°C. Britton–Robinson universal buffer solution with various pH were prepared and used for the pH titration. All aqueous buffered solutions were freshly prepared and stored in refrigerator (used within one week). A concentrated stock solution of the oxazolidine form (3 mM) in DMSO was prepared. Aliquot of the stock solution was added quantitatively to the aqueous buffer solutions of various pH to obtain the final concentration of the probe of 3 μM. The solutions were adequately stirred before spectral measurements. UV/vis as well as fluorescence pH titrations were recorded in triplicate and the outcomes were expressed as the mean ± standard deviation.

Cyclic Stability Test

OH-CyBr (3 μM) was dispersed in Britton–Robinson buffer at pH=12.0. The solution pH was alternately adjusted between 2 and 12, and fluorescence intensities at 423 nm and 657 nm were recorded after each adjustment. Four pH cycles were performed to evaluate the reversibility and stability of the probe between acidic and alkaline

conditions.

Preparation of OCBr

The procedure is as follows. DSPE-MPEG2000 (10 mg) was dissolved in H_2O (10 mL) and stirred at room temperature for 30 min. Separately, OH-CyBr (5 mg) was dissolved in THF (1 mL). Then, 0.5 mL of the OH-CyBr/THF solution was added to 5 mL of the above DSPE-MPEG2000 solution, causing the mixture to turn blue. Under sonication (100 W) using a cell disruptor, TEA was slowly added dropwise until the solution became light yellow, followed by further sonication for 5 min. The mixture was then dialyzed against deionized water for 4 h to obtain the nanoformulation.

Release of C-CyBr

The in vitro release behavior of C-CyBr from OCBr nanoparticles was evaluated using a dialysis method. Briefly, OCBr nanoparticle solution (5 mL) was placed into a dialysis bag (molecular weight cutoff, MWCO: 3500 Da) and immersed in 10 mL of buffer solution at pH 6.5 or pH 7.4 under gentle stirring at 37 °C in the dark. At predetermined time intervals, 2 mL of the release medium was withdrawn and replaced with an equal volume of fresh buffer to maintain sink conditions. The amount of released C-CyBr was quantified by high-performance liquid chromatography (HPLC) based on a pre-established calibration curve. All experiments were performed in triplicate, and the cumulative release was calculated accordingly.

Singlet oxygen detection (ROS) ***in vitro***

2’,7’-dichlorodihydrofluorescein (DCFH) was used as the probe to detect generation of ROS in water. The concentration of DCFH. The generation of total ROS was quantified by monitoring the fluorescence increase of the 2’,7’-dichlorodihydrofluorescein (DCFH) compound at 525 nm. The concentrations of DCFH were maintained a 5 μM. The concentration of OH-CyBr used was 5 μM. (660 nm, 10 mW/cm^2, 10 s). Superoxide radical $\cdot O_2^-$) generation was measured by the increase in fluorescence of dihydrorhodamine 123 (DHR123) at 525 nm. The concentration of DHR123 was 5 μM. The concentration of OH-CyBr used was 5 μM. (660 nm, 20 mW/cm^2, 30s).

Cell lines selection

Both MCF-7 and 4T1 are malignant cell lines derived from mammary tissue. During the experiments, cell line selection was prioritized according to project’s specific need for cell numbers. The well controlled proliferation of 4T1 makes it ideal for common evaluation models, such as MTT and CLSM analyses. The fast-proliferating nature of 4T1 cells was selected for experiments demanding large quantities of cells, including flow cytometry assay and Establishment of Tumor-Bearing Mice.

Analysis of intracellular uptake

The intracellular uptake was firstly evaluated by cell fluorescence imaging using confocal laser scanning microscopy (CLSM). Typically, 4T1 cells were seeded into the laser confocal cell-culture dish. After 24 h incubation, the medium was replaced by fresh medium containing 3 μM OCBr. Then the cells were incubated for different times

and imaged with CLSM (excitation wavelength E_x=640 nm, emission wavelength E_m = 650-750 nm).

Cytotoxicity experiments

The 4T1 cells, MCF-7 cells and A549 cells were seeded at a density of 10^4 cells per well in 96-well plate and incubated at 37 ºC for 24 h. Then the original medium was removed, and the cells were treated with OCBr nanoparticles at varying concentrations for 4 h. The light groups need irradiated (660 nm, 20 mW/cm^2) for 10 min and further incubated for 24 h at 37 ºC. Subsequently, the cells were rinsed and added 3-(4,5-dimethylthiazol-2-yl)-2,5-diphenyl tetrazolium bromide MTT solution (5 mg/mL) to each well. After the cells were incubated for 4 h, the solution was carefully removed and added 100 μL DMSO.

The absorbance was measured using a microplate reader (SpectraMax M2e) at 450 nm and the cell viability is expressed as a percentage of the controlled population by the following equation:

$$Cell\ viability\ (\%) = \frac{OD_{ps} - OD_{blank}}{OD_{control} - OD_{blank}} \times 100\%$$

Where OD_{ps} represents the absorbance of the therapeutic group, OD_{blank} represents the absorbance of the blank control group (without cells), and $OD_{control}$ represents the absorbance of the control group.

Intracellular ROS detection by DCFH-DA and dihydroethidium (DHE)

The fluorescent probe DCFH-DA (Solarbio, China) was used as an intracellular ROS indicator. Specifically, 4T1 cells were seeded in 35 mm confocal dishes at a density of 1×10^4 cells/mL and cultured for 24 h. The cells were then divided into four groups and incubated under different conditions: PBS, PBS + Light, OCBr and OCBr + Light. After 10 min of incubation, the old medium was discarded, and the cells were further cultured in fresh medium containing 10 μM DCFH-DA for 30 min. Subsequently, the cells were irradiated with 660 nm light (20 mW/cm^2) for 5 min, and the green fluorescence was captured using a confocal laser scanning microscope (CLSM, E_x=488 nm, E_m=500-550 nm).

By the same method, the production of intracellular superoxide anion radical was detected using DHE. Finally, the red fluorescence was captured via CLSM, E_x=532 nm, E_m=580-630 nm).

ROS scavenging activity assay

4T1 cells were seeded in 35 mm confocal dishes at a density of 1×10^4 cells/mL and cultured for 24 h. The cells were then divided into four groups and incubated under different conditions: PBS, OCBr, OCBr + Light, OCBr + Vc + Light. After 10 min of incubation, the old medium was discarded, and the cells were further cultured in fresh medium containing 10 μM DCFH-DA for 30 min. Add 0.5mM of Vitamin C (Vc) to the OCBr + Vc + Light group before irradiation. Subsequently, the cells were irradiated with 660 nm light (20 mW/cm^2) for 5 min, and the green fluorescence was captured

using a confocal laser scanning microscope (CLSM, E_x=488 nm, E_m=500-550 nm).

Co-localization of subcellular organelles

The subcellular distribution of OCBr was verified by CLSM and commercial subcellular organelle dyes (Hoechst 3334 (E_x = 405 nm and E_m = 400-500 nm), LysoTracker Green (E_x = 488 nm and E_m = 500-600 nm) or MitoTracker Green (E_x = 488 nm and E_m = 500-600 nm)). 4T1 cells were seeded in 35 mm confocal dishes at a density of 1×10^4 cells/mL and cultured overnight in the medium. Afterwards, fresh medium containing 3 μM OCBr (E_x = 640 nm, E_m = 650-750 nm) and the corresponding commercial dyes was added to each culture dish, followed by incubation in the incubator for 30 min. Then, CLSM was used to observe and collect the fluorescence images of the cells, and the Pearson correlation coefficient was calculated.

Calcein-AM/PI cytotoxicity assay

As for Calcein-AM/PI staining, 4T1 cells were seeded in 35 mm confocal dishes at a density of 1×10^4 cells/mL and cultured for 24 h. The cells were then divided into four groups and incubated under different conditions: PBS, PB + Light, OCBr and OCBr + Light (660 nm, 20 mW/cm^2, 20 min). Then, the old medium was discarded, and the cells were further cultured in fresh medium containing 5 μM each of Calcein-AM and PI for 30 min. The green fluorescence signals of Calcein-AM and red fluorescence signals of PI ware imaged by a CLSM (green channel: E_x = 488 nm and E_m = 500-540 nm, red channel: E_x = 532 nm and E_m = 560-600 nm).

Lysosomal Disruption Assay

Acridine orange (AO) was used to evaluate the lysosomal disruption induced by OCBr NPs in 4T1 cells. The cells were seeded in 35 mm confocal culture dishes and incubated at 37 °C with 5% CO_2 for 24 h. The cells were divided into four groups and treated under different conditions: PBS group, PBS + light group, OCBr group, and OCBr + light group (660 nm, 20 mW/cm^2, 20 min). After incubation for 4 h, the light groups were exposed to irradiation. Following the respective treatments, the cells were incubated with 5 μM AO for 30 min. After staining, the cells were washed three times with PBS and replenished with fresh culture medium. Fluorescence images were acquired using CLSM to detect the green and red fluorescence of AO (green channel: E_x = 488 nm, E_m = 500-540 nm; red channel: E_x = 561 nm, E_m = 590-650 nm).

HMGB-1 and CRT cellular immunofluorescence staining assay

Cells were seeded in confocal dishes at a density of 1×10^5 cells cm^{-2} and cultured for 24 h. After cultivation, the cells were washed three times with PBS, followed by the addition of OCBr (5 μM) for incubation. 30 min later, the cells were irradiated with a 660 nm (20 mW cm^{-2}). Then, the confocal dishes were washed 2–3 times with PBS, with each wash lasting 3–5 min. The cells were fixed with 4% paraformaldehyde at 25 °C for 20 min; after fixation, they were rinsed with PBS (3–5 min per rinse). 100% Triton X-100 (abbreviated as Tri) was diluted to 1% with PBS, and the confocal dishes were treated with this diluted solution for permeabilization (25–30 min); post-

permeabilization, the cells were washed with PBS (3–5 min per wash). Blocking was performed using 1% bovine serum albumin (BSA) or 5% fetal bovine serum (FBS). Primary antibodies against CRT and HMGB1, diluted at a ratio of 1:1000, were added to the confocal dishes. The cells were either incubated at 4 °C overnight or at 37 °C for 1–2 h; after incubation, the dishes were washed with PBS (3–5 min per wash). Fluorescent secondary antibodies, diluted at a ratio of 1:1000, were added to the confocal dishes, and the cells were incubated at room temperature for 1 h in the dark. Thereafter, the dishes were washed with PBS (3-5 min per wash). 4',6-diamidino-2-phenylindole (DAPI), diluted at a ratio of 1:2000, was added for staining. Ten min later, imaging was conducted using a confocal microscope (E_x = 488 nm, E_m = 500-540 nm).

Animals and tumor model

This study was conducted in accordance with the Guide for the Care and Use of Laboratory Animals published by the US National Institutes of Health (8th edition, 2011). The female BALB/c mice aged about 4-5 weeks were purchased from Liaoning Changsheng biotechnology Co. Ltd. The animal protocol was approved by the local research ethics review board of the Animal Ethics Committee of the Dalian University of Technology (DUT20230822). The subcutaneous tumor model was established by subcutaneously injecting 50 μL of 5×10^6 4T1 cells suspended in PBS into the right hind leg of the mouse. Tumors that reached an approximate volume of 100 mm^3 were deemed suitable for experimental use. The tumor volume (V) of 4T1 tumor-bearing mice was calculated using the formula:

$$V = \frac{ab^2}{2}$$

a: mouse tumor length; b: mouse tumor width.

***In vivo* fluorescence imaging**

To study the enrichment of nanoparticles in tumors, the 4T1 tumor-bearing mice were injected with OCBr (1 mg mL^{-1}, 100 μL) in the tail vein, and then observed the *in vivo* fluorescence signals at the different post-injection time (2, 6, 10, 14, 18, 22, 48 h) by using the small animals imaging system (NightOWL II LB983 live imaging system, E_x= 640 ± 10 nm, E_m= 700 ± 10 nm).

***In vivo* antitumor evaluation**

The mice were randomly divided into 4 groups: 1) the PBS group of mice administrated with PBS (100 μL) alone; 2) the PBS + Light group of mice administrated with PBS and irradiated with 660 nm irradiation (50 mW cm^{-2}) for 10 min; 3) the group of mice administrated with OCBr (1 mg mL^{-1}, 100 μL) alone; 4) the OCBr + Light group of mice administrated with OCBr (50 μM) and irradiated with 660 nm irradiation (50 mW cm^{-2}) for 10 min. The light irradiation was performed after 22 h injection after the injection of PBS or OCBr. The tumor growth and body weight change were monitored every 2 days. The tumors and major organs (e.g., hearts, livers, lungs, spleens and

kidneys) were harvested at the end of antitumor studies for immunofluorescence examination and H&E staining.

TUNEL and Ki67 assay

The excised tumors were preserved in a 4% formaldehyde solution, subsequently embedded in paraffin, and subjected to TUNEL and Ki67 staining for analysis. The paraffin-embedded tumor samples were sectioned to a thickness of approximately 8.0 μm using a rotary microtome (model YD-1508A) and subsequently stained with 0.25% toluidine blue O. DNA fragmentation was labeled utilizing a TUNEL apoptosis detection kit. Ki67 staining assays, serving as indicators of cell proliferation, were conducted in accordance with established protocols. Embedding and TUNEL/Ki67 staining were done by Liaoning Jijia Biotechnology Co., Ltd. Antibodies were acquired from Liaoning Jijia Biotechnology Co., Ltd.

In vivo biosafety assay

The biosafety assay was conducted through the assessment of mice body weight, and histological examination using hematoxylin and eosin (H&E) staining. After 16 days post-treatment, the mice were euthanized, and harvested main organs for histological analysis by means of H&E staining.

In Vivo antitumor immune activation

16 days after treatment, tumors and spleens were harvested from mice in different treatment groups. Tumor tissues were cut into small pieces, digested with a mixed enzyme solution (5.0 mL of cell culture medium containing collagenase IV and collagenase I) at 37°C for 1 h, ground, and then centrifuged to obtain single-cell suspensions. After grinding and centrifugation of the spleens, red blood cells were removed using red blood cell lysis buffer, and single-cell suspensions were obtained by centrifugation again. Subsequently, the cell suspensions were filtered through a nylon cell strainer, supplemented with 1% fetal bovine serum, stained with fluorescently labeled antibodies, and detected by flow cytometry (Invitrogen, Thermo Fisher Scientific, AFC2). For dendritic cell maturation analysis, single-cell suspensions were stained with anti-CD11c-FITC, anti-CD80-APC, and anti-CD86-PE antibodies; for T cell activation analysis, cell suspensions were stained with anti-CD45-FITC, anti-CD3-APC, anti-CD4-PE, and anti-CD8a-PE antibodies. -

Statistical analysis

All the experimental data were performed at least three independent measurements ($n \geq 3$). *In vivo* therapeutic experiments were performed five independent measurements. The data in this paper were expressed as the mean ± standard deviations (SD). The data in this paper were expressed as the mean ± standard deviations (SD). Statistical analysis and data processing, including one-way ANOVA and Student's t-test, were performed using Origin 2019. $p < 0.05$ indicates a significant difference. Significant difference was defined as $^*p < 0.05$, $^{**}p < 0.01$, and $^{***}p < 0.001$.

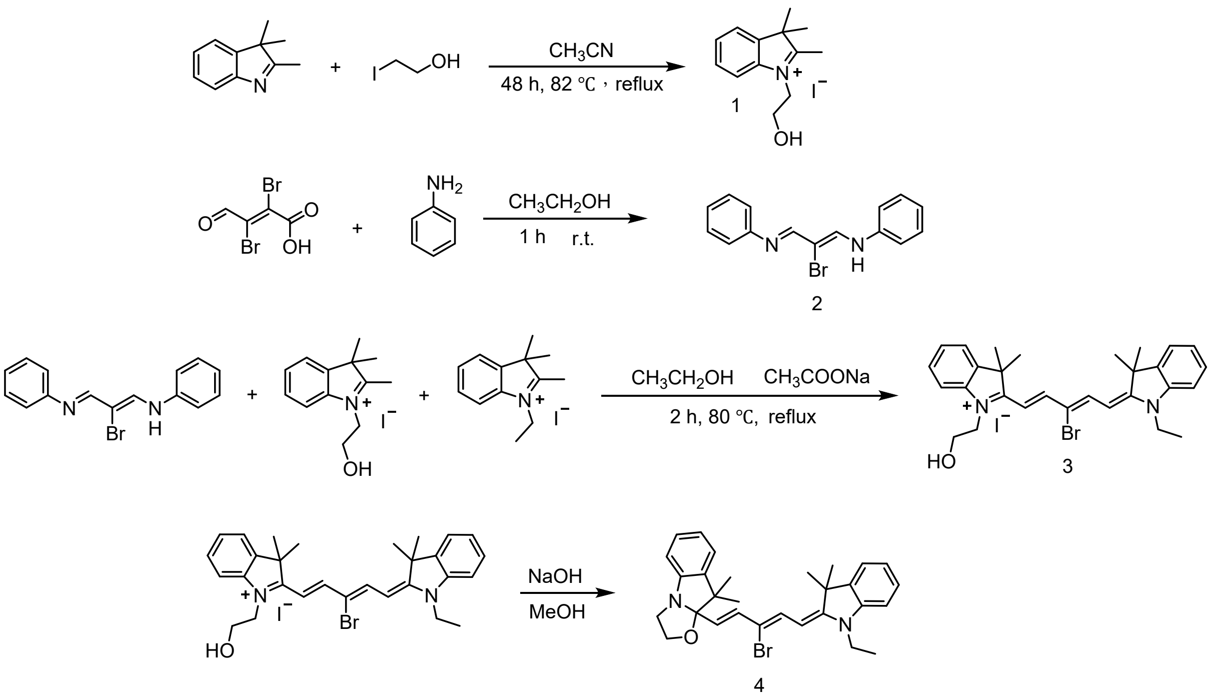

Scheme S1. The synthesis process of C-CyBr.

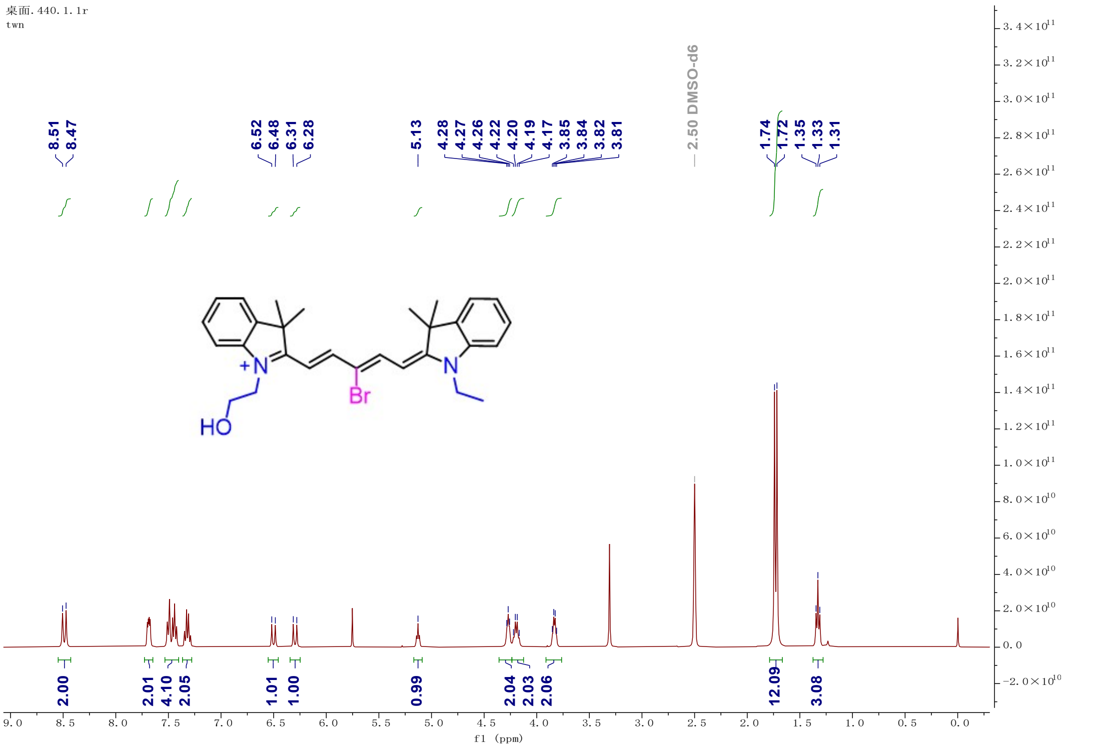

Figure S1. ^{1}H NMR spectrum of OH-CyBr (400 MHz, DMSO-d_6).

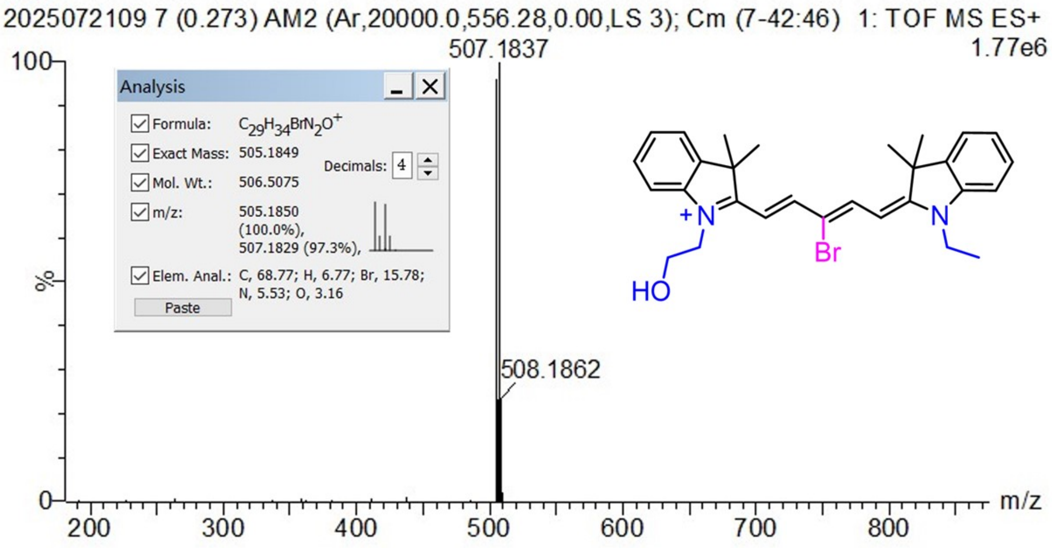

Figure S2. ESI-MS spectrum of OH-CyBr.

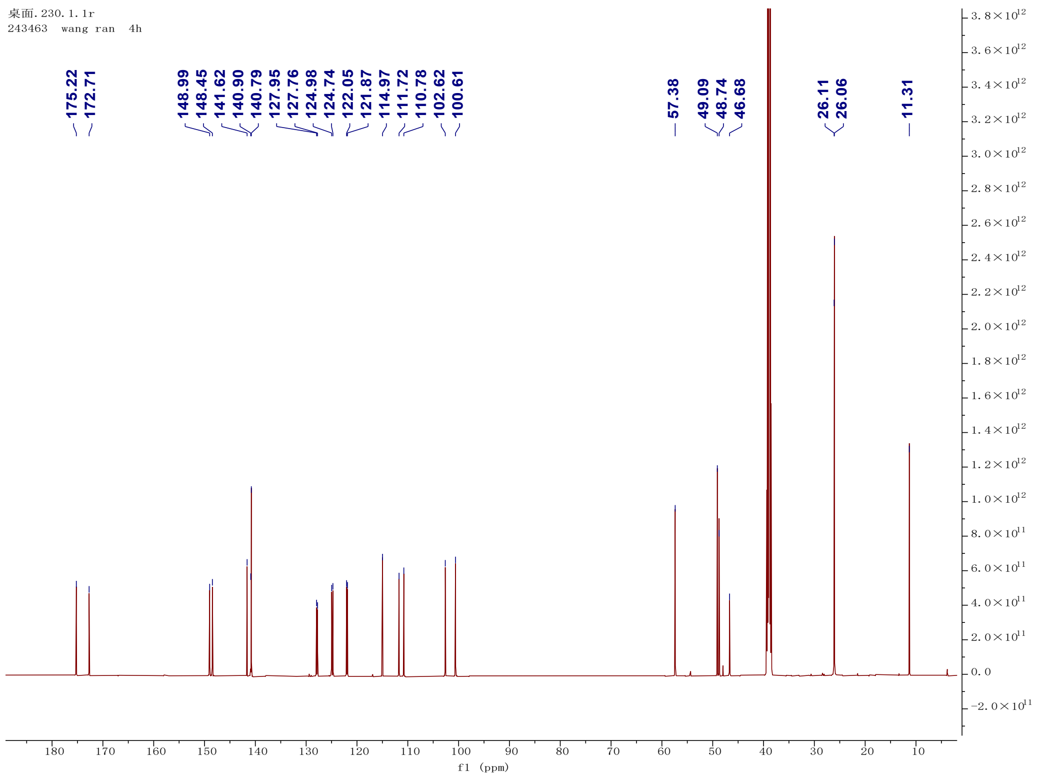

Figure S3. ^{13}C NMR spectrum of C-CyBr (400 MHz, DMSO-d$_6$).

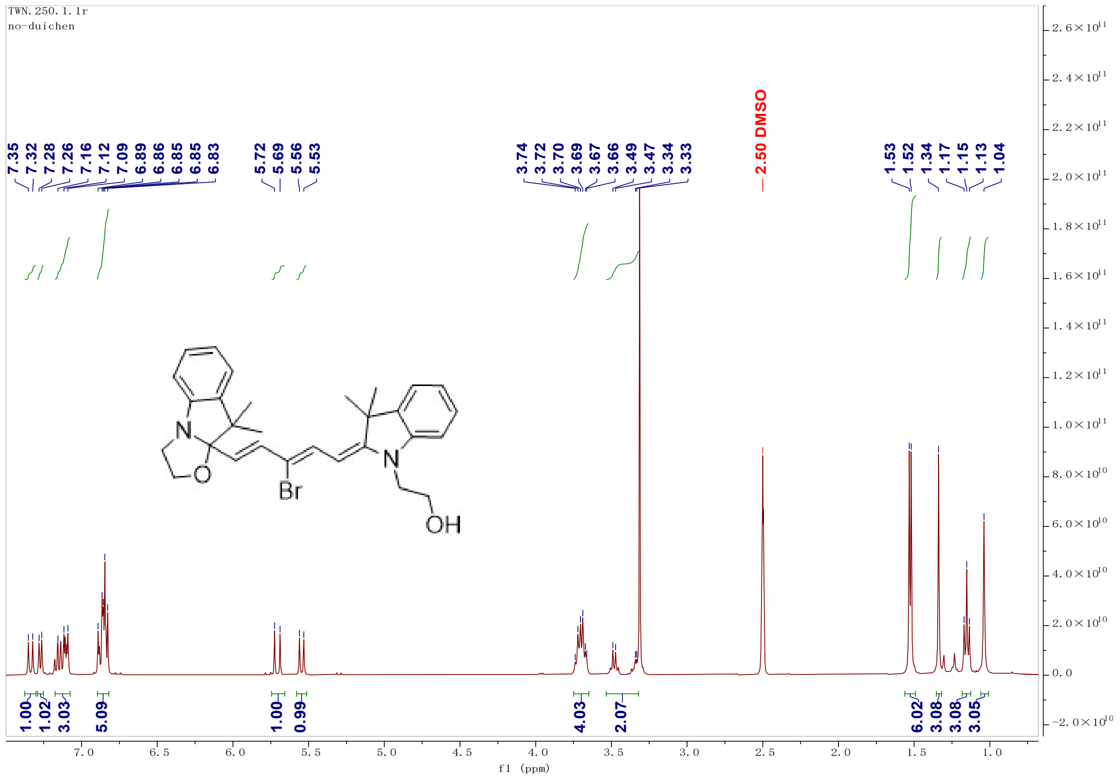

Figure S4. ^{1}H NMR spectrum of C-CyBr (400 MHz, DMSO-d_6).

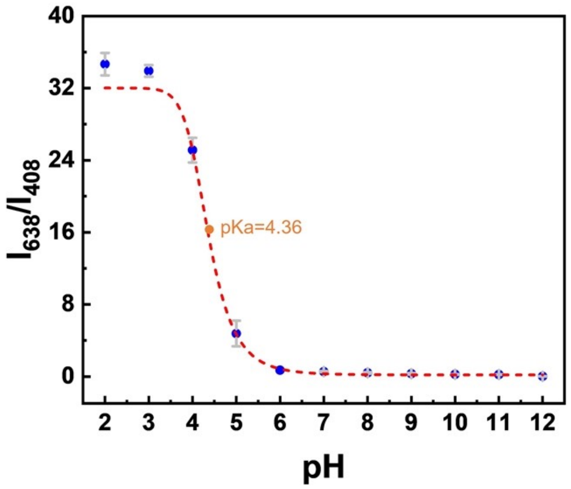

Figure S5. Plot of absorbance ratio (638 nm / 408 nm) of the photosensitizer at different pH values.

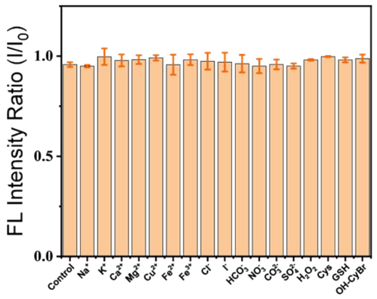

Figure S6. No significant fluctuations are observed in the fluorescence signal of the probe (2.5 μM) at 635 nm in the presence of various cations. 10 mM Na^+, 10 mM K^+ and 200 μM for Ca^{2+}, Mg^{2+}, Cu^{2+}, Fe^{2+}, Fe^{3+}, 200 μM for Cl^- , I^- , HCO_3^- ,HCO_3^-, NO_3^-, CO_3^{2-}, SO_4^{2-} , and bioactive small molecules like glutathione (GSH, 5 mM), cysteine (Cys, 5 mM), and H_2O_2 (1 mM) under physiological conditions (37 °C, pH 7.4).

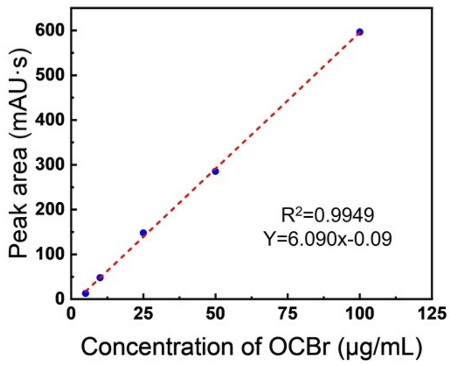

Figure S7. HPLC calibration curve of OCBr showing the linear relationship between peak area and concentration.

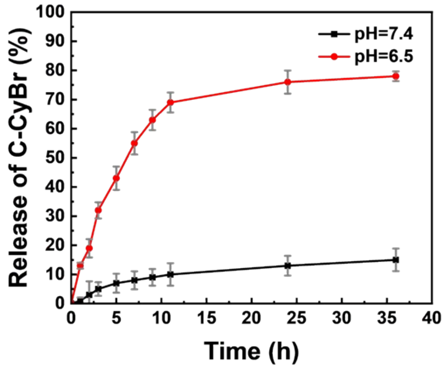

Figure S8. In vitro drug release profiles of OCBr at pH 6.5 and pH 7.4

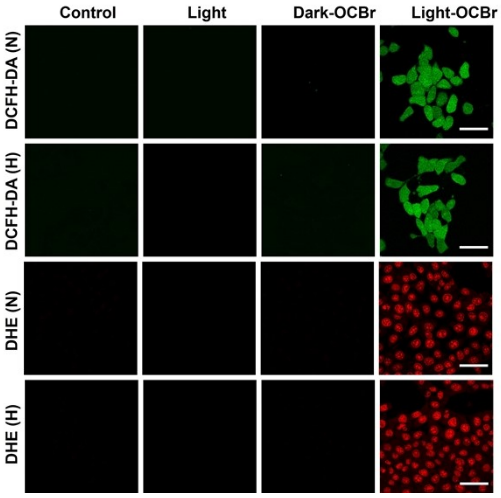

Figure S9. OCBr induces ROS generation in 4T1 cells under normoxic and hypoxic conditions. Cells were incubated with 2 μM OCBr, irradiated, and stained with DCFH-DA (for total ROS) or DHE (for superoxide anion, $\cdot O_2^-$) before confocal imaging. Scale bar: 20 μm.

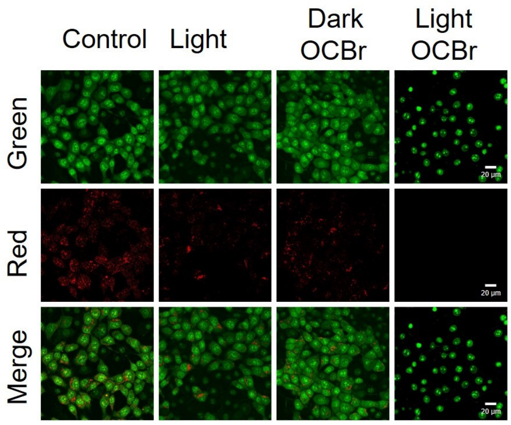

Figure S10. Fluorescence imaging related to lysosomal apoptosis in different treatment groups. Scale bar: 20 μm.

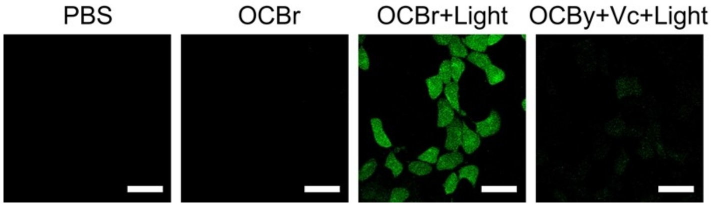

Figure S11. Inverted fluorescence microscopy images of ROS generation in 4T1 cells under different treatment conditions. Green fluorescence from DCFH-DA indicates the presence of ROS. Scale bar: 40 μm.

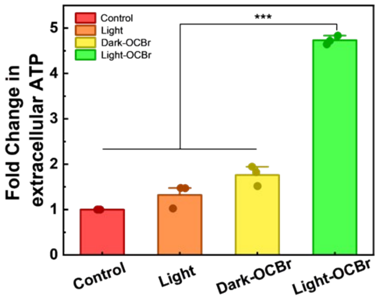

Figure S12. ATP was secreted by 4T1 cells following 24 h of incubation with OCBr. Statistical significance was determined via two-tailed Student's t-test (***p ≤ 0.001).

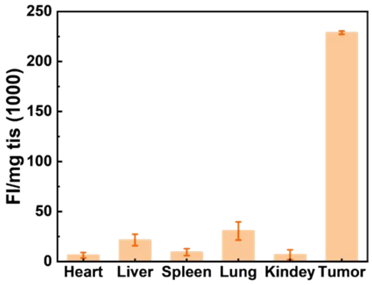

Figure S13. Quantification diagram of relative fluorescence intensity of tumor, heart, liver, spleen, lung and kidney.

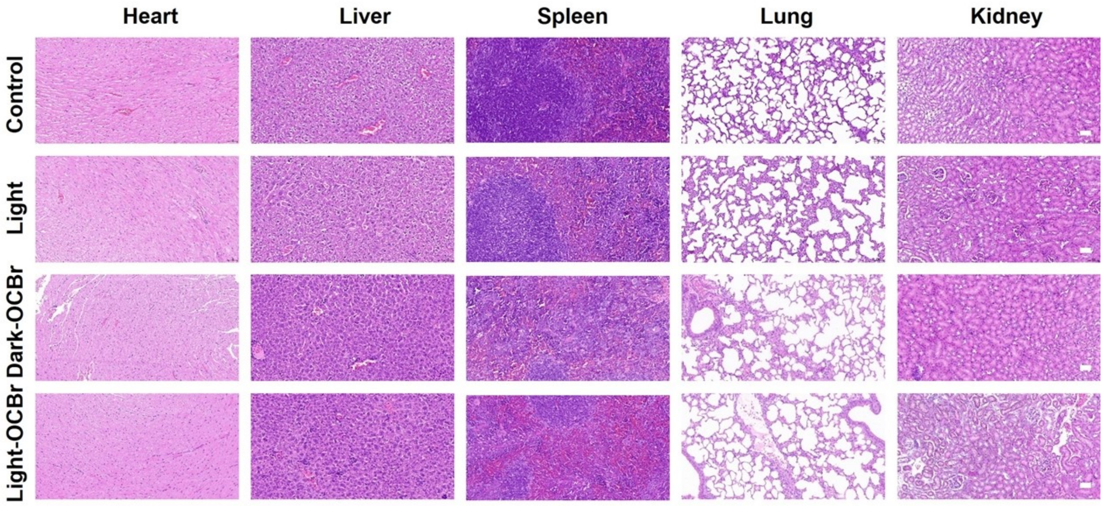

Figure S14. H&E staining of typical organs of mice 30 days after intravenous administration under different treatment conditions. Scale bars: 50 μm.

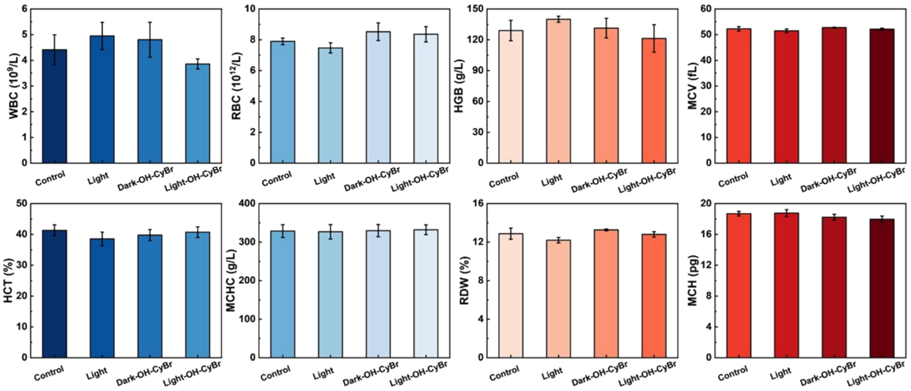

Figure S15. Hematological indices of mice after different treatments: (a) White blood cell count (WBC, $\times 10^9$/L), (b) Red blood cell count (RBC, $\times 10^{12}$/L), (c) Hemoglobin level (HGB, g/L), (d) Mean corpuscular volume (MCV, fL), (e) Hematocrit (HCT, %), (f) Mean corpuscular hemoglobin concentration (MCHC, g/L), (g) Red blood cell distribution width (RDW, %), and (h) Mean corpuscular hemoglobin (MCH, pg). The treatment groups include Control, Light, Dark-OCyBr, and Light-OCBr (the number of experimental replicates n can be supplemented according to actual conditions).

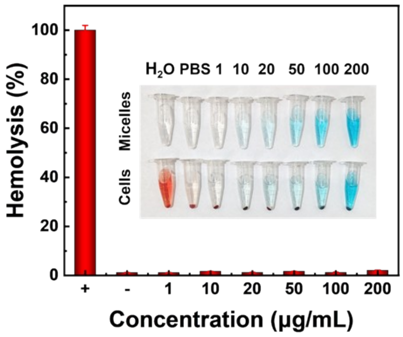

Figure S16. Hemolysis assay of OCBr and its blood compatibility evaluation.
